# Equivalence of Gyn GEC-ESTRO guidelines for image guided cervical brachytherapy with EUD-based dose prescription

**DOI:** 10.1186/1748-717X-8-266

**Published:** 2013-11-13

**Authors:** William Shaw, William ID Rae, Markus L Alber

**Affiliations:** 1 Department of Medical Physics (G68), University of the Free State, Nelson Mandela Drive, Park West, Bloemfontein 9300, South Africa; 2 Department of Oncology, Aarhus University, Nørrebrogade 44/5, Aarhus 8000, Denmark

**Keywords:** Image guided brachytherapy, Planning study, Equivalent uniform dose, Dose volume constraints, Comprehensive volume, Worst case estimate

## Abstract

**Background:**

To establish a generalized equivalent uniform dose (gEUD) -based prescription method for Image Guided Brachytherapy (IGBT) that reproduces the Gyn GEC-ESTRO WG (GGE) prescription for cervix carcinoma patients on CT images with limited soft tissue resolution.

**Methods:**

The equivalence of two IGBT planning approaches was investigated in 20 patients who received external beam radiotherapy (EBT) and 5 concomitant high dose rate IGBT treatments. The GGE planning strategy based on dose to the most exposed 2 cm^3^ (D2cc) was used to derive criteria for the gEUD-based planning of the bladder and rectum. The safety of gEUD constraints in terms of GGE criteria was tested by maximizing dose to the gEUD constraints for individual fractions.

**Results:**

The gEUD constraints of 3.55 Gy for the rectum and 5.19 Gy for the bladder were derived. Rectum and bladder gEUD-maximized plans resulted in D2cc averages very similar to the initial GGE criteria. Average D2ccs and EUDs from the full treatment course were comparable for the two techniques within both sets of normal tissue constraints. The same was found for the tumor doses.

**Conclusions:**

The derived gEUD criteria for normal organs result in GGE-equivalent IGBT treatment plans. The gEUD-based planning considers the entire dose distribution of organs in contrast to a single dose-volume-histogram point.

## Background

Recently, the treatment of cervical cancer has been advanced through the use of image guided brachytherapy (IGBT) [1-4]. The Groupe Européen de Curiethérapie (GEC) and the European SocieTy for Radiotherapy & Oncology (ESTRO) working group (Gyn GEC-ESTRO WG, GGE) presented guidelines that comprise imaging and organ segmentation for planning of every treatment fraction [5,6]; subsequently, limited imaging approaches have been derived [7,8]. Such an approach adapts for organ motion and tumor shape changes by conforming the prescribed dose to the target volume of the day, and thereby increases the chance of applying effective IGBT doses in successive fractions. This image- and volume-based planning strategy allows for a per-fraction analysis of dose distributions and dose volume histograms (DVHs). Further, the total delivered dose up to and including the last treatment fraction can be estimated for clinical target volumes (CTV) and organs at risk (OAR). This constitutes a risk-controlled dose prescription method with DVH criteria for tumor and normal tissue volumes. The relevance of these criteria has been demonstrated by linking them to toxicity [9-11] and local control [11-14]. However, contouring and organ motion are the major contributors of uncertainties in IGBT [15].

The GGE technique requires MRI for tumor and OAR delineation with applicators in-situ. Unfortunately many clinics have limited availability of MRI. One alternative is CT imaging, but due to the lower contrast, CT based planning results in increased OAR volumes, CTV delineation uncertainty and consequently unnecessarily large CTVs, as one tends to plan conservatively [16-19]. These uncertainties can produce lower CTV doses [3,16] if normal tissue DVH criteria are adhered to. At the same time, contour uncertainty leads to uncertainty of derived DVH criteria for toxicity scoring or tumor control and an uncertainty in the addition of OAR and tumor DVHs for obtaining worst-case estimates of the accumulated dose [6,15,20,21]. Furthermore, with the increased use of more conformal external beam radiotherapy (EBT) techniques such as intensity modulated radiotherapy (IMRT), the addition of DVH parameters for such worst case estimates can become unreliable.

This raises the question whether a volume-based treatment plan metric such as the equivalent uniform dose (EUD) [22] could be more robust against contouring and imaging uncertainties than DVH. In EBT planning, the generalized EUD (gEUD) is well established [23-25] and is mathematically equivalent to the DVH reduction scheme of the Lyman-Kutcher-Burman (LKB) normal tissue complication probability (NTCP) model [26-29]. It is our intention to establish a gEUD-based prescription method for IGBT that can replace the original GGE prescription in terms of dose-volume criteria, but offers advantages in terms of safety and robustness against uncertainties. Further, EUD sports favorable mathematical properties that allow a reliable worst-case estimate of the accumulated dose.

We investigate this question with a three-stage planning study of fractionated IGBT. In stage 1, we record the EUD values of OARs achieved with plans obtained from the dose-volume constrained GGE guidelines. From this, we establish corresponding EUD criteria. In stage 2, the treatments are planned according to these EUD constraints, and their safety is assessed according to the GGE DVH criteria. Finally, in stage 3, the full treatments (EBT + 5 fractions IGBT) of both strategies are compared by both metrics.

## Methods

### Patient selection, imaging and contouring

Ethical approval (ETOVS NR 214/09) was received for this study. Twenty patients who had been treated with high dose rate (HDR) IGBT for carcinoma of the cervix between October 2009 and January 2011 were randomly selected (Table 1). All patients received EBT consisting of 25 fractions of 2 Gy via a 4-field box technique without midline shielding, and 5 concomitant IGBT treatment fractions of 4.7 Gy (± 0.8 Gy) to the High Risk CTV (HR-CTV; discussed below) with a standard magnetic resonance imaging compatible tandem-ring (Nucletron®). Intra-uterine source positions were located at 1 cm intervals from the ring to the tip, while the length of the intra-uterine applicator was adapted to tumor extent. Source positions in the ring were fixed for all treatments. Our center’s high workload requires that implantations be done under conscious sedation without vaginal packing. Treatment plans were produced on axial CT images for lack of MRI facilities.

**Table 1:** Patient, volume and treatment characteristics

***Characteristic***	***No of patients and/or value(s)***
*Total nr of patients*	*20*
*Total EBT dose*	*50 Gy*
*Total nr of EBT fractions*	*25*
*Total nr of IGBT fractions*	*5*
*Total IGBT dose (Mean ± SD)*	*4.7 ± 0.8 Gy*
*Total nr of CT datasets in study*	*100*
*FIGO stage (n)*	
*II*	*5*
*III*	*12*
*IVa*	*3*
*Volume in cc (Mean ± SD)*	
*HR-CTV @ 1*^*st*^*IGBT treatment*	*49.0 ± 21.0*
*IR-CTV @ 1*^*st*^*IGBT treatment*	*119.0 ± 43.0*
*Rectum*	*94.8 ± 32.6*
*Bladder*	*108.0 ± 91.6*
*Dose objectives/constraints*	
*HR-CTV D90*	*≥ 85 Gy*
*IR-CTV D90*	*≥ 60 Gy*
*Rectum D2cc*	*≤ 70 Gy*
*Bladder D2cc*	*≤ 80 Gy*

*Abbreviations*: *SD* standard deviation, *cc* cubic centimeters.

Contouring was based on clinical examination and CT images, using the GGE guidelines for the HR-CTV, Intermediate Risk CTV (IR-CTV) and the rectum and bladder walls. The GTV had to be omitted as it cannot be identified on CT images. The HR-CTV consisted of the whole cervix and macroscopic extent of the disease at the time of imaging for IGBT. The IR-CTV encompassed the HR-CTV plus a variable margin depending on the initial extent of the disease, considering tumor regression in response to treatment. The OAR walls and outline with content were delineated according to the same set of recommendations.

### Fractionation and dose evaluation parameters

According to the GGE recommendations we recorded the following parameters for purposes of comparison: Minimal dose received in 0.1, 1, and 2 cc of the maximal dose regions of the OARs (D0.1, 1, 2 cc; outer wall plus content), dose to 90% (D_90_) of the HR- and IR-CTVs, as well as the EUDs of OAR walls and CTVs.

Full DVHs of each treatment fraction were available in the Flexiplan (Nucletron®) treatment planning system and dose was converted to a 2 Gy equivalent dose (EQD2) [30]. According to GGE, the linear quadratic (LQ) model parameters of α/β being 3 Gy for OARs and 10 Gy for tumors (α being 0.3 Gy^-1^) were applied. Since the treatment was concomitant HDR brachytherapy, repair half-times and repopulation were neglected.

The EUD for target volumes was calculated relative to the EBT dose delivered in 2 Gy fractions (d = 2 Gy) from the surviving fraction as:

(1) $$\mathrm{EUD}=\frac{-\mathrm{Log}\left(S\right)}{\alpha +\mathrm{\beta d}}$$

To consider the heterogeneity in dose distributions, the differential DVH of tumors was used to calculate the surviving fraction for each treatment:

(2) $$S=\Sigma_{k}v_{k}S\left(D_{k}\right)$$

*S* is calculated from *D*_*k*_, the dose bin for the *v*_*k*-*th*_ fractional tumor volume.

The gEUD calculation was used for OARs [28,31], again considering a reference dose of 2 Gy per fraction and is given by

(3) $$\mathrm{gEUD}={\left(\Sigma_{k}v_{k}{D_{k}}^{a}\right)}^{\frac{1}{a}}$$

where D_k_ is the EQD2 for the *v*_*k*-*th*_ fractional OAR volume and *a* is the volume effect parameter. The gEUD for rectum and bladder walls was calculated using volume effect parameters (*a*) of 12 for the rectum and 8 for the bladder [23,32,33].

For simplicity we refer to the EUD based, adaptive IGBT planning strategy as the comprehensive volume technique (CV), emphasizing the fact that EUD considers the entire organ volume.

### Study 1: prescription constraints

One possibility to establish the gEUD prescription constraints for IGBT treatment planning is to collect them from literature, another by a planning study. From Söhn et al. (2007), we can choose the gEUD upper limit for the rectum to be 67.8 Gy (3.55 Gy EUD per IGBT fraction), at approximately 10% NTCP for grade II (G2) rectal bleeding. However, in the following this gEUD is verified against a rectum D2cc constraint of 70 Gy EQD2 [9,10] by the planning study. Bladder NTCP model data are scarce and uncertain [34] due to unaccounted variations in filling. Consequently, the bladder gEUD constraint is determined by the planning study. There are bladder dose guidelines based on the GGE work that show more than 5 - 10% late complication rates when the D2cc is in the order of 70–100 Gy EQD2 [9,10,19,35,36].

To derive the bladder wall gEUD dose constraints, the GGE planning strategy was followed to achieve at least 7.0 Gy per fraction (85 Gy EQD2 from EBT and IGBT) to the HR-CTV D90. Treatment plans were produced for each treatment fraction on the 100 CT datasets. Each plan started from the standard loading pattern and was manually or graphically optimized until the HR-CTV dose objective was reached, or until one of the two OAR constraints in question prevented any further CTV dose escalation (Table 1). *Bladder EUD constraints:* HR-CTV dose was further increased beyond the CTV objective until the bladder D2cc criterion was reached. This constraint was chosen as 80 Gy total dose from EBT and IGBT, resulting in 6 Gy EQD2 per IGBT fraction. The procedure was repeated on all plans and for each the associated bladder wall gEUD was computed. Consequently, the bladder wall gEUD is solely determined by the D2cc of the bladder and is not influenced by any other OAR criterion or the CTV doses. *Rectum EUD constraints:* To verify the chosen gEUD of 67.8 Gy for an upper rectal limit, we repeated this constraint derivation procedure for the rectal wall by limiting the total rectum D2cc EQD2 to 70 Gy (4.0 Gy EQD2 per fraction).

All 100 “bladder-limited” plans are maximized to the bladder constraint of 6.0 Gy D2cc per fraction and the corresponding bladder wall gEUDs were recorded and all 100 “rectum-limited” plans are maximized to the rectum dose constraint of 4.0 Gy D2cc and the associated rectum wall gEUDs were recorded. From these data, the variation in bladder and rectal wall gEUD at fixed DVH criteria could be found and the EUD criteria could be derived or verified from these gEUD frequency distributions.

### Study 2: safety of EUD constraints in terms of GGE constraints

To test the safety of the CV technique, we investigated the appropriateness of the bladder- and rectum wall EUD constraints in terms of the GGE dose volume criteria. Here we maximized the same dose distribution as in study 1 for each treatment plan, but to the point where the bladder wall gEUD constraint was reached instead of the D2cc constraint. At this point we recorded the corresponding D2cc (and other DVH parameters). This procedure was repeated for the rectal wall by maximizing dose to the rectum gEUD constraint. Thus a single plan was optimized against each of the organs at risk separately.

### Study 3: comparison of GGE and CV planning strategies for the entire treatment

Once the robustness of the CV technique in each fraction has been established, the two planning strategies can be compared in terms of OAR and CTV dose for a full treatment. The GGE based plans for each patient and each fraction adhered to the two OAR D2cc constraints (Table 1) per fraction, whichever was met first. The HR-CTV D90 was targeted to be at least 85 Gy in total. No upper CTV constraints were set and dose was maximized until an OAR D2cc constraint was reached. The total dose from IGBT and EBT was calculated. For the CV technique the OAR EUD constraints were employed that were found earlier. Finally, the two strategies could be compared in terms of D90, D2cc and EUD.

## Results

### Prescription constraints

**Figure 1:**
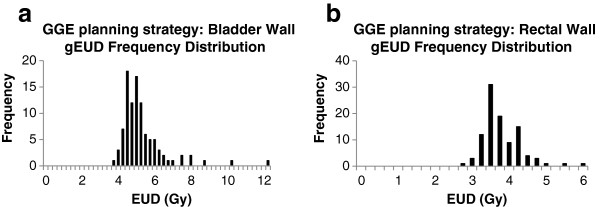
**GGE planning strategy: Bladder and rectal wall EUD frequency distributions.** Frequency distributions of bladder **(a)** and rectum **(b)** wall EUDs when dose is maximized to 6 Gy D2cc for bladder and 4 Gy D2cc for rectum.

The frequency distributions of the OAR wall EUDs for bladder-limited and rectum-limited plans are displayed in Figure 1. Table 2 provides a summary of the statistics. The spread of EUDs results from the fact that the gEUD is calculated from the full OAR DVH while D2cc is limited to a small volume. Furthermore, the D2cc volume may often include organ contents. Notice further some extreme outliers, which are a consequence of an unfavorable organ location in some fractions that brings large parts of the organ close to the high dose range, but below the D2cc criterion.

**Table 2:** Summary of the statistical parameters of the gEUD variations with D2cc and EUD criteria

***Statistical measure***	***Dose (Gy) GGE strategy***	***Dose (Gy) CV strategy***
*Bladder D2cc/gEUD* constraint *(planning)*	*6.00*	*5.19*
*Bladder Wall gEUD/D2cc*		
*Mean*	*5.19/6.00*	*5.19/6.25*
*SD*	*1.25/0.00*	*0.00/1.01*
*Bladder D0.1 cc*		
*Mean*		*9.97*
*SD*		*0.85*
*Bladder D1cc*		
*Mean*		*7.21*
*SD*		*0.98*
*Rectum D2cc/gEUD* constraint *(planning)*	*4.00*	*3.55*
*Rectum Wall gEUD/D2cc*		
*Mean*	*3.67/4.00*	*3.55/3.96*
*SD*	*0.53/0.00*	*0.00/0.49*
*Rectum D0.1 cc*		
*Mean*		*5.80*
*SD*		*0.29*
*Rectum D1cc*		
*Mean*		*4.46*
*SD*		*0.44*

*Abbreviations*: *SD* standard deviation.

The average gEUD of the rectal wall at a D2cc constraint of 4.0 Gy was 3.67 Gy (±0.53 Gy) which is comparable to the 3.55 Gy from our external beam rectum EUD constraint choice. If this average gEUD was reached in all of the 5 fractions, the NTCP would be ranking at approximately 11%. The average bladder gEUD at a D2cc constraint of 6.0 Gy was 5.19 Gy (±1.25 Gy). The values: rectum wall gEUD ≤ 3.55 Gy and bladder wall gEUD ≤ 5.19 Gy were established as the upper limits for the CV technique. Thus, the total EUD constraint for the bladder wall equals 75.95 Gy.

### Safety of EUD criteria in terms of GGE criteria

**Figure 2:**
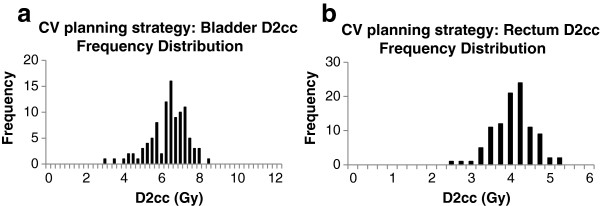
**CV planning strategy: Bladder and rectal D2cc frequency distributions.** Frequency distributions of the bladder **(a)** and rectum **(b)** D2ccs when expanding the dose distribution to 5.19 Gy EUD for bladder and 3.55 Gy EUD for rectum.

The safety of these EUD criteria was verified by comparing the D2cc values of CV plans with those obtained from the GGE strategy. Figure 2 shows the distribution of D2cc for the OARs with the EUD criteria as determined in the previous section, while Table 2 compares the D2cc statistics of the frequency distributions. Figure 2 shows that the D2cc distributions are skewed towards lower values and show no outliers towards high doses. The mean of the D2cc distributions closely resembles the GGE criteria, see Table 2. For a fractionated treatment, the EUD criteria can thus be considered safe, because even in the worst case (the same organ is dose-limiting in all fractions) the sum of the D2cc of n fractions is likely to be smaller or equal to n times the mean D2cc of the distributions, due to their left-skew. Since the choice of EUD criteria is somewhat arbitrary, we identified those levels, gEUD(x), that result in no more than x% of the 100 treatment plans exceeding the associated GGE criterion, see Table 3.

**Table 3:** Different gEUD(x) levels resulting in percentage x of treatment fractions with D2cc larger than the GGE constraint and mean and standard deviations of the resulting distributions

***x % of treatment fractions***	***Rectum gEUD(x) (Gy)***	***Rectum mean D2cc ± SD (Gy)***	***Bladder gEUD(x) (Gy)***	***Bladder mean D2cc ± SD (Gy)***
***10***	*3.12*	*3.49 ± 0.43*	*4.22*	*5.11 ± 0.81*
***25***	*3.35*	*3.74 ± 0.46*	*4.48*	*5.42 ± 0.86*
***48***	*3.55*	*3.96 ± 0.49*		
***50***	*3.58*	*3.99 ± 0.50*	*4.86*	*5.87 ± 0.94*
***70***			*5.19*	*6.25 ± 1.01*

*Abbreviations*: *SD* standard deviation.

We have also found very good correlations between D0.1 cc and D2cc for the rectum (R^2^ = 0.84), as well as excellent correlation between D1cc and D2cc for the rectum (R^2^ = 0.96). This means that if D2cc can be controlled via the use of the EUD, ulcerations, fistulas and rectal bleeding will also be controlled. Similarly, we have found excellent correlation between bladder D1cc and D5cc with D2cc (R^2^ = 0.95 and R^2^ = 0.93 respectively), and a worse correlation between D0.1 cc and D2cc (R^2^ = 0.63).

### Comparison of GGE and CV planning strategies

**Figure 3:**
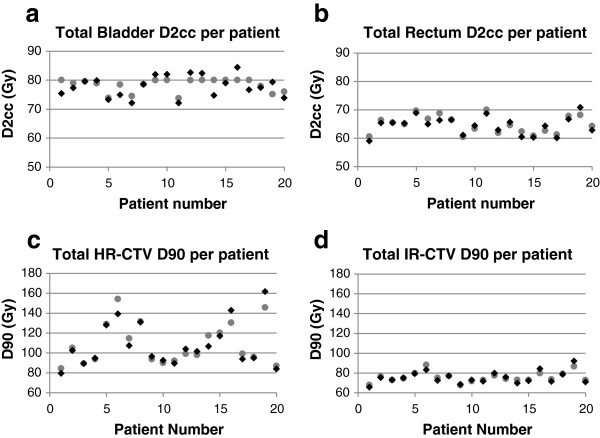
**Total DVH parameters per patient.** Total EQD2 for bladder **(a)** and rectum **(b)**, and D90 for the HR- **(c)** and IR-CTV **(d)**. Data are shown for the GGE technique (circles) and the CV technique (diamonds).

**Figure 4:**
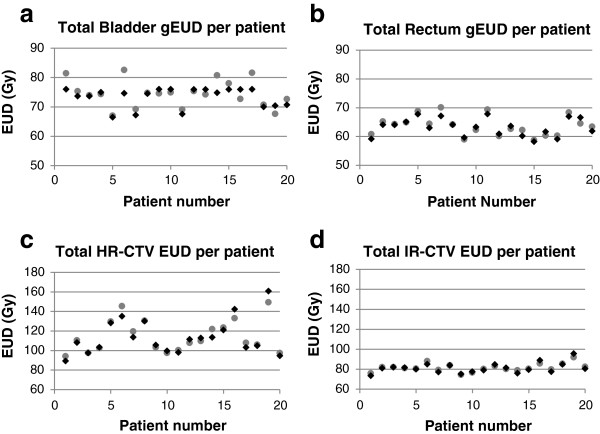
**Total EUD per patient.** Total EUD for bladder **(a)** and rectal wall **(b)**, HR- **(c)** and IR-CTV **(d)**. Data are shown for the GGE technique (circles) and the CV technique (diamonds).

The two planning approaches were compared in terms of total dose from all 5 IGBT fractions plus the EBT component for the patients in the study. Very similar total dose parameters for the two techniques were found. Figure 3 displays the total dose in the two planning techniques for rectum and bladder D2cc, and HR- and IR-CTV D90. Figure 4 displays the rectal and bladder wall gEUDs, and the HR- and IR-CTV EUDs. Table 4 provides the average and standard deviations of their frequency distributions, indicating very similar means.

**Table 4:** Summary of the statistical variations of the DVH parameters and EUD variations over the full treatment course

***Statistical measure***	***Technique***	***Rectum***	***Bladder***	***HR-CTV***	***IR-CTV***
		***D2cc***	***D2cc***	***D90***	***D90***
*Mean (Gy)*	*GGE*	*64.85*	*78.29*	*108.49*	*75.85*
*SD*		*3.10*	*2.29*	*20.59*	*5.22*
*Mean (Gy)*	*CV*	*64.51*	*77.87*	*107.77*	*75.36*
*SD*		*3.20*	*3.70*	*21.95*	*6.16*
		***gEUD***	***gEUD***	***EUD***	***EUD***
*Mean (Gy)*	*GGE*	*63.66*	*74.53*	*114.28*	*81.51*
*SD*		*3.42*	*4.58*	*16.40*	*4.12*
*Mean (Gy)*	*CV*	*63.18*	*73.32*	*113.58*	*81.19*
*SD*		*3.07*	*3.31*	*17.90*	*5.07*

All values in Gy.

*Abbreviations*: *SD* standard deviation.

## Discussion

We have established OAR gEUD criteria for IGBT treatments that are very comparable to those obtained from the GEC-ESTRO guidelines. EUD constraints can thus be considered a safe and efficient alternative to D2cc criteria.

Compared to a D2cc constraint, which considers an isolated small volume, gEUD has the advantage to consider the dose distribution in the OAR comprehensively and still give high doses a large weight, especially if the volume effect parameter a is significantly greater than 1. For the same reason, it is also less sensitive to contouring and may therefore be a more robust choice if MRI is not available for IGBT planning. To see this, assume that contouring errors lead to errors in the volume of the dose bins of the DVH. Applying the laws of error propagation, we find that the error in D2cc is proportional to the inverse slope of the DVH at D2cc (which tends to be shallow in BT) and proportional to the volume error at that dose bin. In contrast, the error in gEUD is both proportional to the weighted root-mean-square of the volume errors in the dose bins (thus less dependent on a single bin) and smaller by a factor 1/a. This ties in with the intuition, that any kind of average over a number of uncertain quantities (such as EUD) is less uncertain than any single one of these quantities.

The derived EUD criteria depend on the reference D2cc criteria and the volume effect parameter *a*. Since gEUD is a power-law function of dose, it scales with the same factor as D2cc. Small deviations from this law are caused by the EQD2 correction. Within reason, our criteria can therefore be calibrated to different fractionation schemes, i.e. scaled by the ratio of the desired D2cc versus the value used here.

The volume effect parameters (a = 8 for bladder, a = 12 for rectum) are derived from the literature. They do express a very small volume effect of the complications in question, which is also the implicit rationale behind the D2cc criterion. We confirm that the influence of the choice of *a* on our results is small, although safer when *a* ≥ 8, since D2cc becomes increasingly smaller with a large *a* at fixed constraint levels; see *a* value variance in Table 5. It is thus considered safe to err towards large *a* values, i.e. smaller volume effect, when the exact value is not known.

**Table 5:** Variation of gEUD and D2cc for different values of the gEUD volume parameter

***Volume parameter (a)***	Rectum gEUD constraint (Gy)*	***Rectum D2cc (Gy)***^***#***^	Bladder gEUD constraint (Gy)**	***Bladder D2cc (Gy) ***^***##***^
*8*	*3.09 ± 0.37*	*4.66 ± 0.52*	*5.19 ± 1.25*	*6.25 ± 1.01*
*9*	*3.26 ± 0.42*	*4.43 ± 0.51*	*5.56 ± 1.44*	*5.89 ± 1.00*
*10*	*3.41 ± 0.46*	*4.24 ± 0.50*	*5.89 ± 1.61*	*5.60 ± 0.99*
*11*	*3.54 ± 0.50*	*4.09 ± 0.50*	*6.19 ± 1.77*	*5.35 ± 0.97*
*12*	*3.67 ± 0.53*	*3.96 ± 0.49*	*6.46 ± 1.91*	*5.15 ± 0.96*
*13*	*3.78 ± 0.56*	*3.85 ± 0.49*	*6.72 ± 2.04*	*4.98 ± 0.95*
*14*	*3.88 ± 0.59*	*3.75 ± 0.48*	*6.95 ± 2.16*	*4.83 ± 0.93*
*15*	*3.97 ± 0.61*	*3.67 ± 0.48*	*7.17 ± 2.26*	*4.70 ± 0.92*
*16*	*4.06 ± 0.63*	*3.60 ± 0.47*	*7.36 ± 2.36*	*4.59 ± 0.91*

*Calculated with a 4.0 Gy rectum D2cc constraint.

**Calculated with a 6.0 Gy bladder D2cc constraint.

^#^Calculated with a 3.55 Gy (a = 12) rectum gEUD constraint.

^##^Calculated with a 5.19 Gy (a = 8) bladder gEUD constraint.

Occasionally, the use of EUD criteria for IGBT is safer than D2cc. Observe the outliers in Figure 1 which are caused by rare unfavorable organ geometries that bring a lot of the organ volume close to the high dose region. In contrast, EUD criteria do not produce excessive D2cc values because of their mathematical construction, which gives very high weights to sub-volumes with a high dose. From Table 2, the average D2cc for the OARs, when dose is maximized to each OAR’s gEUD constraint, is virtually the same as the GGE-D2cc that was used to derive the EUD criteria. Although there is some dispersion of D2ccs around this average, none of the D2ccs were found to be unacceptably high. If the EUD constraints are reduced, as shown in Table 3, to decrease D2cc constraint violations, small changes in EUD result in large reductions in D2cc and a smaller variance of D2cc. Our results suggests that a 6 to 8% reduction in OAR gEUDs produce more than 25% fewer treatment plans that could violate a D2cc constraint. Since we know that D0.1 cc and D1cc also correlates well with D2cc, CV plans that control D2cc would subsequently control the resultant D0.1 cc and D1cc DVH parameters as well.

The D2ccs of the CV technique are evaluated against data from other studies in Table 6, which includes D0.1 and D1cc endpoints. The comparison shows that maximizing OAR dose to the EUD constraints does not result in OAR over-dosage. The total average bladder and rectal D0.1, D1 and D2cc when OAR dose is maximized to the EUD constraints falls in a lower range than those presented by Georg et al. for LENT/SOMA scores of 1–4 and VRS scores of 3–5 [9]. The population averages in their studies [9,10] are comparable to the dose levels in this study. We have also found that especially the rectal doses in this study are in the lower range of toxicity rates for G2-4 side effects. Based on the Georg et al. studies [9,10,35], our criteria relate to a probability of finding G2-G4 side effects in the range of 5-10%.

**Table 6:** Summary of the average DVH parameters in total dose (Gy) of the CV treatment technique, compared to other published values

***DVH parameter***	***CV***	***Georg et al. *** [9]	***Georg et al. *** [10]	***Levitchi et al. *** [33]	***Jürgenliemk-Schulz et al. *** [38]	***Jürgenliemk-Schulz et al. *** [39]	***Nesvacil et al. *** [40]	***Lindegaard et al. *** [41]
***Method***	*HDR*	*HDR*	*HDR*	*PDR*	*PDR*	*HDR/PDR*	*HDR*	*PDR*
***Rectum***								
*D0.1 cc*	*79 ± 1*	88 ± 10*	*83 - 132*^*a*^	*83*^*b*^				
		81 ± 13**	86 ± 27**	65 ± 15**				74 ± 9**
*D1cc*	*72 ± 2*	76 ± 7*	*71 - 87*^*a*^					
		70 ± 9**	69 ± 14**					69 ± 6**
*D2cc*	*70 ± 2*	72 ± 6*	*67 - 78*^*a*^	*68*^*b*^				
		66 ± 8**	65 ± 12**	57 ± 8**	66 ± 6**	54 ± 2**^c^	57 ± 6**	67 ± 6**
						69 ± 2**^d^		
***Bladder***								
*D0.1 cc*	*100 ± 3*		*61 - 178*^*a*^	*109*^*b*^				
			162 ± 75**	78 ± 22**				86 ± 12**
*D1cc*	*86 ± 3*		*71 - 116*^*a*^					
			108 ± 31**					77 ± 8**
*D2cc*	*81 ± 3*		*70 – 101*^*a*^	*72*^*b*^	81 ± 6**	53 ± 2**^c^	76 ± 9**	
			95 ± 22**	64 ± 11**		101 ± 11**^d^		73 ± 6**

*Abbreviations*: *SD* standard deviation, *HDR* high dose rate, *PDR* pulsed dose rate.

*Clinical symptoms (LENT/SOMA) score 1–4 and Rectoscopic changes (VRS) score 3–5.

**Population average; no interstitial needles.

^a^5% - 10% probability of G2-G4 side effects (dose range not shown).

^b^Approximately 10% probability of G2-G4 toxicity (dose range not shown).

^c^Small volume tumor.

^d^Large volume tumor.

These dose endpoints are also very comparable with studies where large HR-CTV volumes were investigated and no interstitial needles were used. As shown in the study of Jürgenliemk-Schulz et al. [36], we expect that interstitial needles would decrease the EUD of OARs in large tumor volume cases as well. For bladder, we found good correspondence with the results of Levitchi et al. [37], Jürgenliemk-Schulz et al. [36,38], Nesvacil et al. [39] and Lindegaard et al. [40]. Since there were no upper dose boundaries for the CTV, the CTV dose is expected to spread widely, driven solely by the OAR geometries and relative positions. From Figures 3 and 4 it is clear that the CV technique does not result in under-dosage of the CTVs.

An important aspect of gEUD is, that it allows an easy worst-case estimate of the gEUD of the total accumulated treatment dose by virtue of Jensen’s inequality [41,42]. The sum of EUDs of each treatment fraction is always greater or equal (for OARs; smaller or equal for targets) to the EUD of the sum of the fraction doses:

(4) $$\text{EUD}\left(E\left[\tilde{D}\right]\right)\leq E\left[\text{EUD}\left(\tilde{D}\right)\right]=E\left[\text{EUD}\left(D\right)\right]$$

where E[] is the sum over all fractions, $\tilde{D}$ is the dose of each fraction, warped to reference geometry, and D the dose as computed for the patient geometry of the particular fraction. Hence, the left hand side is the EUD of the properly accumulated total dose, while the right hand side is the sum of the EUDs as computed for each fraction individually. For target volumes, the inequality reverses. This estimate is of particular importance for pelvic radiotherapy, where deformable registration of images is difficult to perform reliably. Hence, EUD addition gives a worst case scenario for OARs and CTV without the need for deformable image registration and dose warping [42].

D2cc is not a convex function of dose and is not additive in a strict sense, so that further assumptions about the dose distribution have to be made. Jensen’s inequality also applies to maximum and minimum dose, so that, if D2cc and D90 have a strong correlation to the former, the inequality holds for the latter approximately “by proxy”. The versatility of EUD summation as worst case estimate extends to the addition of very heterogeneous OAR EBT doses, for example lymph node boosts. Finally, because there is a variability in reported dose-volume cut-offs for OARs in IGBT [9,35,37,43] and these also differ from cut-offs in EBT, EUD is helpful in combining the experience in both areas and relating it to the LKB model [44]. Conversely, documented brachytherapy toxicity rates can be useful for focused dose escalation in EBT, for example dose painting.

## Conclusions

Concluding, a GEC-ESTRO-like IGBT plan adaption is feasible with EUD criteria, instead of D2cc criteria. Because of the mathematical construction of gEUD, and the fact that it considers the organ volume comprehensively, it is inherently more robust against contouring uncertainties. This could make gEUD a better choice than D2cc if IGBT has to be performed on CT, instead of MR, images. The summation of EUDs per treatment fraction gives a reliable worst case estimate of the total treatment dose, which opens possibilities for safe dose escalation in IGBT or simultaneous integrated boost in EBT.

## Competing interests

The authors declare that they have no competing interests.

## Authors’ contributions

WS contributed to study design, data acquisition and management, treatment planning, physical evaluation of treatment plans, result analysis and writing the final manuscript. WIDR contributed to result analysis and drafted the manuscript. MLA contributed to the conception of the study, study design and revision of the manuscript. All authors read and approved the final manuscript.
